# Enhancing Cardiovascular Risk Prediction in Type 2 Diabetes: The Role of NT-proBNP as a Prognostic Biomarker

**DOI:** 10.7759/cureus.92997

**Published:** 2025-09-23

**Authors:** Abdullah Hassan, Omar Alfauri, Ayesha Ashraf, Laiba Nadeem, Komal Zaman, Arsalan P Dalal, Fatima Raja, Nabeel Al-Omari, Ishtiaq Ahmad, Usman Safdar, Muhammad Zauraiz Malik

**Affiliations:** 1 Internal Medicine, King Edward Medical University, Lahore, PAK; 2 Internal Medicine, Misr University for Science and Technology, Giza, JOR; 3 Medicine, King Edward Medical University, Lahore, PAK; 4 Medicine, Punjab Medical College, Faisalabad, PAK; 5 Medicine and Surgery, Acharya Shri Chander College of Medical Sciences and Hospital, Jammu, IND; 6 Clinical Sciences, Yarmouk University, Irbid, JOR; 7 Internal Medicine, Khyber Medical College, Peshawar, PAK; 8 Medicine and Surgery, King Edward Medical University, Lahore, PAK

**Keywords:** biomarker, cardiovascular, heart failure, n-terminal pro-b-type natriuretic peptide, nt-probnp, type 2 diabetes

## Abstract

Cardiovascular complications in type 2 diabetes remain a significant clinical challenge, with existing risk prediction approaches potentially missing important prognostic information. N-terminal pro-B-type natriuretic peptide (NT-proBNP), a biomarker reflecting cardiac stress and dysfunction, may offer valuable insights for enhanced risk stratification in this population. This systematic review examined the available evidence regarding the role of NT-proBNP in cardiovascular risk prediction among patients with type 2 diabetes. Following comprehensive literature searches across multiple databases and systematic screening processes, four studies involving over 8,000 participants were identified for qualitative synthesis. Across all studies, elevated NT-proBNP levels demonstrated consistent associations with increased cardiovascular risk. The biomarker exhibited strong discriminative capabilities for various cardiovascular endpoints, with performance metrics suggesting utility comparable to complex multivariable clinical models when used independently. When incorporated alongside traditional risk factors, NT-proBNP consistently enhanced predictive performance across studies. Cross-sectional investigations revealed a notable prevalence of subclinical cardiac dysfunction among asymptomatic diabetes patients, indicating potential opportunities for early identification and intervention. However, several important limitations constrain the current evidence base, including the small number of available studies, heterogeneity in methodological approaches, variation in biomarker thresholds, and absence of data demonstrating clinical benefit from biomarker-guided care strategies. Despite these constraints, NT-proBNP appears to hold promise as a valuable adjunct for cardiovascular risk assessment in type 2 diabetes populations. The ability of this marker to identify subclinical dysfunction and predict adverse outcomes beyond conventional clinical variables addresses important gaps in current risk stratification approaches. Nevertheless, the limited evidence necessitates further investigation through large-scale prospective studies and randomized controlled trials to establish standardized diagnostic thresholds, validate predictive performance across diverse populations, and demonstrate that biomarker-enhanced risk assessment translates into meaningful improvements in clinical outcomes and healthcare delivery.

## Introduction and background

Type 2 diabetes mellitus (T2DM) is a major global health challenge, characterized by chronic hyperglycemia resulting from insulin resistance, progressive β-cell dysfunction, or both. According to the International Diabetes Federation, the prevalence of diabetes is projected to rise from an estimated 537 million adults in 2021 to 783 million by 2045, with T2DM accounting for over 90% of cases worldwide [1]. The cardiovascular system is profoundly affected in this population, with atherosclerotic cardiovascular disease (ASCVD) and heart failure (HF) representing the leading causes of morbidity and mortality in individuals with T2DM [2]. Despite advances in pharmacological therapy and preventive strategies, residual cardiovascular risk remains high, underscoring the need for improved tools for risk stratification beyond traditional clinical variables.

Conventional cardiovascular risk assessment models, such as the Framingham Risk Score, UK Prospective Diabetes Study (UKPDS) risk engine, and American College of Cardiology/American Heart Association pooled cohort equations, incorporate factors like age, sex, blood pressure, lipid profile, smoking status, and duration of diabetes [3]. While these models have been validated in large populations, their predictive accuracy in patients with T2DM is suboptimal, particularly for HF outcomes and composite endpoints [4]. This limitation arises partly from the fact that traditional models primarily capture atherosclerotic risk and do not adequately account for subclinical myocardial stress, fibrosis, or diastolic dysfunction, pathophysiological processes that are highly prevalent in T2DM and contribute substantially to adverse cardiovascular events [5].

In this context, biomarkers reflecting myocardial injury, stress, or remodeling have gained attention as potential adjuncts to established risk prediction models. Among these, the N-terminal pro-B-type natriuretic peptide (NT-proBNP) has emerged as a promising candidate for this purpose. NT-proBNP is an inactive cleavage fragment of proBNP, secreted predominantly by ventricular cardiomyocytes in response to increased wall stress and volume overload. While BNP and NT-proBNP are both established biomarkers for the diagnosis and prognosis of HF, NT-proBNP has superior analytical stability, has a longer plasma half-life, and is less susceptible to rapid clearance, making it advantageous in both clinical and research settings [6]. The potential utility of NT-proBNP in T2DM lies in its ability to refine risk stratification and guide targeted preventive interventions. Incorporating NT-proBNP into existing risk models may improve discrimination (the ability to correctly rank individuals by risk), calibration (agreement between predicted and observed risk), and reclassification (assigning individuals to more appropriate risk categories) [7].

Despite promising findings, several uncertainties remain. NT-proBNP levels are influenced by age, sex, renal function, body mass index, and atrial fibrillation, necessitating careful interpretation in diverse patient populations [8]. Moreover, heterogeneity exists in study designs, assay methodologies, outcome definitions, and statistical approaches used to assess incremental predictive value. Some studies have questioned whether improvements in risk prediction metrics translate into meaningful clinical benefit, particularly in primary prevention settings. Addressing these gaps requires a comprehensive review of the literature to evaluate the strength, consistency, and applicability of the evidence. Given the high cardiovascular burden in T2DM and the limitations of existing risk prediction models, there is a compelling need to explore novel biomarkers that can enhance prognostic accuracy. NT-proBNP, by reflecting underlying myocardial stress and subclinical cardiac dysfunction, holds promise as a valuable adjunct in risk assessment. This systematic review aims to (1) summarize the evidence on the association between NT-proBNP levels and cardiovascular outcomes in individuals with T2DM, (2) evaluate the incremental predictive value of NT-proBNP when added to conventional risk models, and (3) identify knowledge gaps and directions for future research. By synthesizing the current evidence, this review seeks to inform both clinical practice and research priorities in the prevention of cardiovascular complications in T2DM.

## Review

Materials and methods

This systematic review was conducted in accordance with the Preferred Reporting Items for Systematic Reviews and Meta-Analyses (PRISMA) 2020 guidelines to ensure transparency and rigor in study identification, screening, eligibility assessment, and data extraction [9].

Search Strategy

A comprehensive literature search was performed across four major electronic databases from January 2020 to July 2025: PubMed, Embase, Scopus, and Cochrane Library. The search strategy employed a combination of Medical Subject Headings (MeSH) and free-text keywords to capture relevant studies. Search terms included "NT-proBNP", "N-terminal pro-B-type natriuretic peptide", "brain natriuretic peptide", "natriuretic peptide", "type 2 diabetes", "diabetes mellitus", "T2DM", "cardiovascular outcomes", "cardiovascular events", "heart failure", "myocardial infarction", "stroke", "cardiovascular mortality", "risk prediction", "prognosis", and "biomarker". Boolean operators such as "AND" and "OR" were used to combine the terms appropriately. The search strategy was tailored for each database to optimize sensitivity while maintaining specificity. To enhance the comprehensiveness of the review, reference lists of all included studies and related systematic reviews were manually screened to identify any relevant articles that may have been missed in the initial database search. Grey literature sources and conference abstracts were also searched to minimize publication bias.

Eligibility Criteria

We included peer-reviewed studies that investigated the association between NT-proBNP levels and cardiovascular outcomes in individuals diagnosed with T2DM. Eligible study designs included randomized controlled trials (RCTs), prospective and retrospective cohort studies, case-control studies, and cross-sectional studies with prognostic components. Studies were included if they reported any relevant cardiovascular outcomes such as cardiovascular mortality, all-cause mortality, myocardial infarction, stroke, HF hospitalization, composite cardiovascular endpoints, or measures of cardiovascular risk prediction improvement (e.g., C-statistic, net reclassification index, integrated discrimination improvement). We excluded animal studies, case reports, case series, reviews, editorials, conference abstracts without full-text availability, and studies not published in English. Studies that examined BNP rather than NT-proBNP were excluded unless they provided comparative data. Additionally, studies focusing exclusively on T1DM, gestational diabetes, or mixed populations without separate analysis for T2DM were excluded. Only studies involving adult patients (≥18 years of age) with a confirmed diagnosis of T2DM were considered.

Study Selection

All retrieved records were imported into Rayyan software (Rayyan Systems Inc., Cambridge, MA, USA) for duplicate removal and screening management. Following de-duplication, two independent reviewers screened the titles and abstracts of all remaining studies for potential eligibility using predefined inclusion and exclusion criteria. The full texts of studies deemed relevant or potentially relevant were then assessed independently by the same reviewers against the detailed eligibility criteria. Discrepancies in selection were resolved through consensus discussion and, if required, adjudication by a third reviewer. A PRISMA flow diagram was used to document the selection process, including specific reasons for exclusion at each stage.

Data Extraction

Data extraction was conducted independently by two reviewers using a predefined and piloted data extraction form designed specifically for this review. The following variables were extracted: first author, year of publication, country of study, study design, sample size, patient demographics (age, sex, diabetes duration, comorbidities), cardiovascular outcomes assessed, measures of discrimination and calibration (C-statistic, net reclassification index, integrated discrimination improvement), hazard ratios or odds ratios with confidence intervals, and key findings related to incremental predictive value. Where available, information on NT-proBNP cutoff values, subgroup analyses, and adjustment for confounding variables was also extracted. Discrepancies in data extraction were resolved by consensus and, if necessary, by referral to a third reviewer.

Quality Assessment

The methodological quality of included studies was assessed independently by two reviewers using the Newcastle-Ottawa Scale (NOS), evaluating selection of study groups, comparability of groups, and ascertainment of outcome. The quality assessment considered factors such as representativeness of the study population, adequacy of follow-up, completeness of outcome data, appropriateness of statistical analysis, and potential for bias. Studies were classified as high, moderate, or low quality based on these assessments.

Data Synthesis

Due to the anticipated heterogeneity in study designs, patient populations, NT-proBNP measurement methods, outcome definitions, follow-up duration, and statistical approaches, a quantitative meta-analysis was deemed inappropriate. Instead, a comprehensive qualitative narrative synthesis was undertaken. Extracted outcomes were systematically organized and summarized in tabular format according to study characteristics, patient demographics, NT-proBNP measurement details, cardiovascular outcomes assessed, and measures of incremental predictive value. Key trends, consistencies, and discrepancies among studies were identified and discussed. Where possible, subgroup observations based on patient characteristics, diabetes duration, or specific cardiovascular outcomes were noted. Limitations of individual studies and identified gaps in the literature were documented as part of the synthesis to inform future research directions.

Results

Study Selection Process

**Figure 1:**
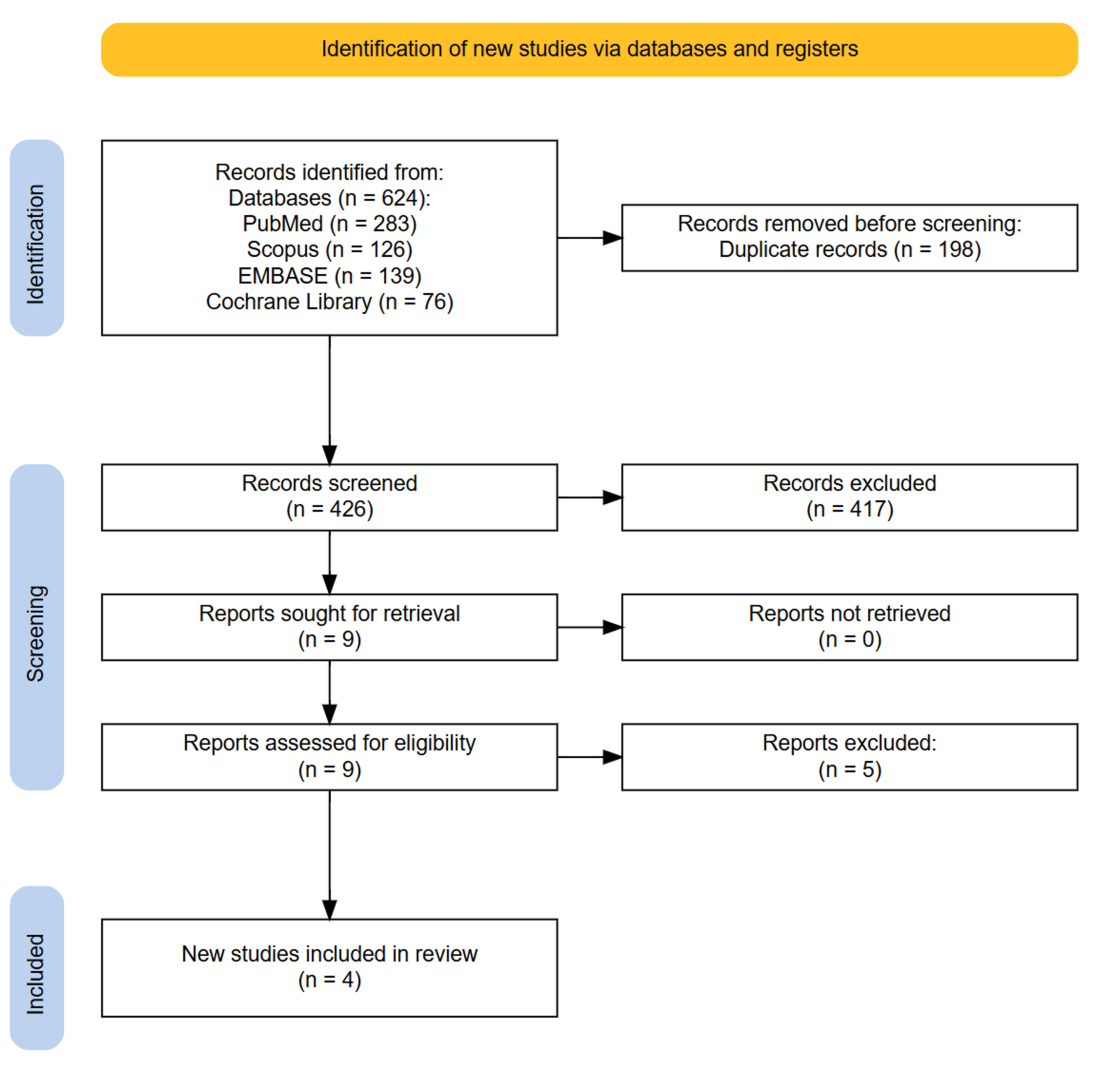
PRISMA diagram illustrating the study selection process.

The comprehensive literature search across four databases yielded a total of 624 records (PubMed: 283; Scopus: 126; Embase: 139; Cochrane Library: 76). After removing 198 duplicates, 426 records underwent title and abstract screening. Following the exclusion of 417 articles, 9 full-text articles were assessed for eligibility. Ultimately, four studies met all inclusion criteria and were included in the qualitative synthesis. These comprised two cross-sectional studies, one prospective cohort study, and one post hoc analysis of an RCT. No additional relevant studies were identified through manual screening of reference lists, indicating comprehensive database coverage (Figure 1).

Study Characteristics

The four included studies encompassed a total of 8,013 participants with T2DM, with sample sizes ranging from 192 to 5,509 patients. Studies were conducted across diverse geographical regions: one from Italy [10], one from China [11], one from India [12], and one multinational study [13]. The mean age of participants ranged from 59 to 70.3 years, with male predominance in most studies (59.8%-67.5%). Mean diabetes duration varied considerably, from newly diagnosed patients to those with a long-standing disease (mean: 11.3 years) [11]. Study designs varied significantly: two cross-sectional analyses examining prevalence and associations, one prospective cohort with a median 5.1-year follow-up, and one post hoc analysis of the ALTITUDE trial with a 2.6-year median follow-up. Primary outcomes included heart stress prevalence based on NT-proBNP thresholds, incident cardiorenal events, mortality and cardiovascular composite outcomes, and left ventricular diastolic dysfunction detection. NT-proBNP measurement was standardized across studies using established laboratory methods, though cutoff values varied from 125 pg/mL to age-adjusted thresholds based on current guidelines (Table 1).

**Table 1:** Characteristics of the studies included in this systematic review. AF: atrial fibrillation, ALTITUDE: Aliskiren Trial in Type 2 Diabetes Using Cardiovascular and Renal Disease Endpoints, BMI: body mass index, BP: blood pressure, CAD: coronary artery disease, CHF: congestive heart failure, CI: confidence interval, CKD: chronic kidney disease, CV: cardiovascular, CVCO: cardiovascular composite outcome, CVD: cardiovascular disease, eGFR: estimated glomerular filtration rate, EF: ejection fraction, HbA1c: hemoglobin A1c (glycated hemoglobin), HDL: high-density lipoprotein, HF: heart failure, HFA-ESC: Heart Failure Association of the European Society of Cardiology, HS: heart stress, LVDD: left ventricular diastolic dysfunction, MACE: major adverse cardiovascular events, MI: myocardial infarction, NT-proBNP: N-terminal pro-B-type natriuretic peptide, OR: odds ratio, RCT: randomized controlled trial, SGLT2i: sodium-glucose cotransporter-2 inhibitor, GLP1-RA: glucagon-like peptide-1 receptor agonist, T2DM: type 2 diabetes mellitus.

Author	Year	Study type	Sample size	Demographics	Aims and objectives	Outcomes assessed	Main findings	Conclusions
Landolfo et al. [10]	2024	Cross-sectional	192 consecutive outpatients	Mean age, 70.3 ± 7.8 years; 67.5% males; 63.8% obese (BMI ≥30 kg/m²); 32.1% with chronic kidney disease (eGFR < 60 mL/min/1.73 m²); Mean arterial BP: 138.5/77.0 ± 15.8/9.9 mmHg	To assess the prevalence of "heart stress" (HS) based on NT-proBNP cut points in asymptomatic T2DM patients with hypertension/high-normal BP eligible for SGLT2i and/or GLP1-RA treatment	Primary: prevalence of HS using age-adjusted NT-proBNP cut points according to 2023 HFA ESC Consensus. Secondary: prevalence of overt HF risk. Associated factors with HS	Median NT-proBNP: 96.0 (38.8-213.0) pg/mL. HS prevalence: 28.6% "HS likely," 43.2% "HS not likely" (grey zone), 28.2% "very unlikely HS." HF risk: 16.7% had NT-proBNP compatible with HF likely. Independent risk factors for HS: CKD (OR = 2.8, 95% CI 1.3-6.2, p = 0.012) and number of antihypertensive drugs (OR = 1.8, 95% CI 1.3-2.5, p = 0.001)	Over a quarter of T2DM patients with hypertension/high-normal BP eligible for SGLT2i/GLP1-RA were at risk of cardiac damage (even subclinical) according to NT-proBNP levels. Most would receive an indication for an echocardiogram and specialist referral, allowing early implementation of strategies to prevent/delay progression to advanced cardiac disease and overt HF. BP burden and renal damage were the most relevant risk factors
Ma et al. [11]	2025	Prospective cohort	1,993 individuals with type 2 diabetes	59.8% male; mean age, 61.1 ± 11.0 years; mean diabetes duration, 11.3 ± 8.66 years; Chinese population, 24.7% had elevated NT-proBNP (≥125 pg/mL); median follow-up, 5.1 years	To evaluate whether NT-proBNP can improve risk stratification and prediction of cardiorenal events in T2DM, beyond that provided by clinical risk factors	Incident AF, CAD, CVD, CHF, CKD, 40% drop in eGFR	Elevated NT-proBNP (≥125 pg/mL) associated with three- to fivefold increased risk of cardiorenal events (all p < 0.001). Good discriminative ability: C-index 0.81-0.89 for various endpoints	NT-proBNP demonstrated promising ability to serve as a prognostic marker for a variety of cardiorenal complications in T2DM. Considering NT-proBNP in clinical assessments could potentially help identify high-risk individuals who may benefit from more intensive therapies. The biomarker improved risk stratification beyond that provided by clinical risk factors alone
Ramaprabha et al. [12]	2025	Retrospective cross-sectional study	319 patients (169 diabetic, 150 non-diabetic) with preserved ejection fraction > 60%	Age: 59 years (median) in both groups; diabetic group, 63% male, 37% female; non-diabetic group, 61% male, 39% female	To explore the potential of NT-pro-BNP as an early marker for LVDD in diabetic patients with preserved EF and determine its role in predicting MACE	Primary: NT-pro-BNP levels, LVDD. Secondary: correlation with HbA1c, lipid profiles, glycemic control, hospital stay due to MACE	NT-pro-BNP significantly higher in diabetic vs non-diabetic patients (4567.00 vs 131.30 pg/mL, p = 0.001). Strong positive correlation between HbA1c and NT-pro-BNP in the diabetic group (r = 0.863, p < 0.001). Negative correlation with HDL (r = -0.715, p < 0.001). HbA1c was the strongest predictor of NT-pro-BNP levels (β = 0.869, p < 0.001)	NT-pro-BNP is clinically significant in T2DM with preserved ejection fraction > 60% for detecting left ventricular diastolic dysfunction; combined NT-pro-BNP screening and 2D echocardiogram represents the best predictor of diastolic dysfunction and hospitalization due to MACE in T2DM patients
Malachias et al. [13]	2020	Post hoc analysis of ALTITUDE RCT	5,509 patients	Age: 64.1 ± 9.8 years (survivors) vs 68.1 ± 9.3 years (non-survivors). Sex: 31.1% female. Race: 54.7% White, 37.2% Asian, 2.4% Black. BMI: 29.7 ± 5.9 kg/m². Inclusion: T2DM + CVD/CKD, ≥35 years	To assess the discriminatory ability provided by NT-proBNP by itself for the prediction of both death and cardiovascular composite outcome compared with a multivariable model in patients with T2DM and CVD or/and CKD	Primary: death from any cause. Secondary: cardiovascular composite outcome (CV death, resuscitated cardiac arrest, nonfatal MI, nonfatal stroke, or unplanned HF hospitalization). Follow-up: median 2.6 years	NT-proBNP alone had similar discriminatory ability as 20-variable base model for death (C-statistic: 0.745 vs 0.744, p = 0.95). NT-proBNP alone similar to base model for CVCO (C-statistic: 0.723 vs 0.731, p = 0.37). Adding NT-proBNP improved base model for death (C-statistic: 0.779 vs 0.744, p < 0.001) and CVCO (C-statistic: 0.763 vs 0.731, p < 0.001). 469 patients (8.5%) died and 768 (13.9%) had CVCO during follow-up	In high-risk patients with T2DM, NT-proBNP by itself demonstrated discriminatory ability similar to a multivariable model in predicting both death and cardiovascular events and should be considered for risk stratification

Quality Assessment

The methodological quality of included studies was assessed using the NOS [14]. Given the diversity in study designs, quality assessment criteria were adapted appropriately for cross-sectional and cohort studies (Table 2).

**Table 2:** Quality assessment of included studies using the Newcastle-Ottawa Scale. The Newcastle-Ottawa Scale (NOS) is a tool used to assess the quality of non-randomized studies (case-control and cohort) in systematic reviews and meta-analyses, focusing on selection, comparability, and outcome/exposure [14].

Author	Selection (max: 4)	Comparability (max: 2)	Outcome (max: 3)	Total score	Quality rating
Landolfo et al. [10]	3	1	2	6	Moderate
Ma et al. [11]	4	2	3	9	High
Ramaprabha et al. [12]	2	1	2	5	Low-moderate
Malachias et al. [13]	4	2	3	9	High

The quality assessment revealed significant heterogeneity in methodological rigor. The two prospective studies demonstrated high quality with comprehensive adjustment for confounding variables, standardized outcome assessment, and adequate follow-up periods [11,13]. Both cross-sectional studies had inherent limitations related to their design, with the study by Landolfo et al. [10] showing better methodological quality than that of Ramaprabha et al. [12] due to a larger sample size and more comprehensive statistical analysis [10,12].

Common methodological strengths included standardized NT-proBNP measurement protocols and clear outcome definitions. However, limitations were notable across studies: single-center recruitment in two studies potentially limiting generalizability, cross-sectional designs in half the studies precluding causal inference, and variable adjustment for important confounders such as renal function and comorbidities. The small number of included studies also limits the robustness of evidence synthesis.

Discussion

This systematic review of four studies encompassing over 8,000 patients with T2DM provides important but limited evidence regarding the role of NT-proBNP in improving cardiovascular risk prediction. Despite the small number of studies, consistent patterns emerge that support the potential clinical utility of this biomarker while highlighting significant knowledge gaps requiring further research. The most compelling evidence comes from the prospective cohort of Ma et al., which demonstrated that elevated NT-proBNP (≥125 pg/mL) was associated with three- to fivefold increased risk of cardiorenal events across multiple endpoints [11]. The study achieved impressive discriminative ability with C-indices ranging from 0.81 to 0.89 for various outcomes, suggesting that NT-proBNP provides substantial prognostic information beyond traditional clinical risk factors. This finding is particularly significant given the prospective study design, adequate follow-up period (median 5.1 years), and comprehensive adjustment for confounding variables.

The ALTITUDE post hoc analysis by Malachias et al. provides complementary evidence from a high-risk population with established cardiovascular disease or chronic kidney disease [13]. The remarkable finding that NT-proBNP alone achieved discriminatory ability similar to a comprehensive 20-variable clinical model (C-statistic 0.745 vs 0.744 for death prediction) suggests exceptional efficiency of this single biomarker in capturing cardiovascular risk. When added to the base clinical model, NT-proBNP significantly improved discrimination for both death (C-statistic improvement from 0.744 to 0.779, p < 0.001) and cardiovascular composite outcomes (0.731-0.763, p < 0.001). The clinical implications are further illuminated by the cross-sectional study of Landolfo et al., which revealed that 28.6% of asymptomatic T2DM patients with hypertension had evidence of "heart stress" based on age-adjusted NT-proBNP cut points, with an additional 16.7% demonstrating levels compatible with likely HF [10]. This suggests substantial subclinical cardiac dysfunction in T2DM populations that would be undetected by conventional clinical assessment. The identification of chronic kidney disease (OR = 2.8) and antihypertensive medication burden (OR = 1.8) as independent risk factors for elevated NT-proBNP provides insights into the patient populations most likely to benefit from biomarker screening.

The mechanistic insights provided by Ramaprabha et al., despite methodological limitations, offer important perspectives on NT-proBNP's relationship with metabolic control [12]. The strong positive correlation between HbA1c and NT-proBNP levels (r = 0.863, p < 0.001) and negative correlation with HDL cholesterol (r = -0.715, p < 0.001) suggest that NT-proBNP may serve as an integrative biomarker reflecting both cardiac status and metabolic dysfunction severity. The finding that HbA1c was the strongest predictor of NT-proBNP levels (β = 0.869) supports the concept that glycemic control directly influences myocardial stress, even in patients with preserved ejection fraction.

However, several critical limitations constrain the interpretation and generalizability of these findings. The most significant limitation is the small number of included studies, which prevents robust evidence synthesis and limits our ability to draw definitive conclusions about NT-proBNP's clinical utility. The heterogeneity in study designs, with only two providing prospective data, further constrains the evidence base for causal relationships between NT-proBNP levels and cardiovascular outcomes. The variation in NT-proBNP cutoff values across studies (125 pg/mL fixed threshold vs age-adjusted values) reflects the lack of consensus on appropriate diagnostic criteria for T2DM populations. This heterogeneity complicates clinical implementation and suggests the need for standardized, diabetes-specific reference ranges. Additionally, the geographic diversity of studies, while providing some evidence of generalizability, also introduces potential confounding due to differences in population characteristics, healthcare systems, and treatment patterns [15].

The limited focus on HF outcomes in most studies, while mechanistically appropriate given the biology of NT-proBNP, provides incomplete evidence regarding its utility for predicting atherothrombotic events, which represent a major component of cardiovascular risk in T2DM. Furthermore, none of the studies provided evidence of clinical benefit from NT-proBNP-guided risk stratification, limiting our understanding of whether improved risk prediction translates into better patient outcomes. Despite these limitations, the consistency of findings across diverse populations and study designs provides encouraging evidence for the potential role of NT-proBNP in T2DM risk assessment. The ability of this biomarker to identify subclinical cardiac dysfunction and predict cardiovascular events beyond traditional risk factors addresses a critical gap in current risk stratification approaches for diabetes patients [16].

Future directions

This systematic review is significantly limited by the small number of included studies (n = 4), which constrains the robustness of evidence synthesis and prevents meta-analytical approaches. The heterogeneity in study designs, with only half providing prospective data, limits causal inference regarding the predictive value of NT-proBNP. Additionally, the variation in outcome definitions, follow-up periods, and statistical approaches across studies makes direct comparison challenging and may obscure important differences in the biomarker's performance across different clinical scenarios. The lack of standardized NT-proBNP cutoff values represents a critical knowledge gap requiring urgent attention [17]. Future research should prioritize the development of diabetes-specific reference ranges that account for age, sex, renal function, and diabetes duration. Large-scale prospective studies with diverse populations are needed to establish evidence-based diagnostic thresholds that optimize clinical utility while maintaining cost-effectiveness.

Importantly, none of the included studies provided evidence that NT-proBNP-guided risk assessment leads to improved clinical outcomes or cost-effective care. RCTs examining whether biomarker-guided therapy allocation improves patient outcomes, reduces healthcare costs, or enhances quality of life are essential before widespread clinical implementation [18]. Such studies should also evaluate the optimal integration of NT-proBNP with other risk assessment tools and examine its utility across different stages of diabetes and cardiovascular disease. Future research directions should include validation studies in external populations, development of integrated risk prediction models combining NT-proBNP with other biomarkers and clinical variables, and economic evaluations of biomarker-guided care strategies. The field would also benefit from studies examining temporal changes in NT-proBNP levels in response to therapeutic interventions, which could inform its utility for monitoring treatment response and disease progression in T2DM populations.

## Conclusions

This systematic review provides encouraging but limited evidence supporting the potential role of NT-proBNP in improving cardiovascular risk prediction for patients with type 2 diabetes. The consistent findings across diverse populations suggest that NT-proBNP can identify subclinical cardiac dysfunction and predict cardiovascular events beyond traditional risk factors, with impressive discriminative performance comparable to complex multivariable models. The biomarker's ability to detect "heart stress" in asymptomatic patients and its strong correlation with metabolic control indicators highlight its potential clinical utility. However, significant knowledge gaps remain, including the lack of standardized diabetes-specific reference ranges, limited prospective outcome data, and the absence of evidence demonstrating improved clinical outcomes from biomarker-guided care. Before widespread clinical implementation, large-scale prospective studies and randomized controlled trials are needed to establish optimal diagnostic thresholds, validate predictive performance across diverse populations, and demonstrate that NT-proBNP-guided risk assessment translates into meaningful improvements in patient outcomes and cost-effective healthcare delivery.
